# Analysis of human neuronal cells carrying *ASTN2* deletion associated with psychiatric disorders

**DOI:** 10.1038/s41398-024-02962-4

**Published:** 2024-06-03

**Authors:** Yu Hayashi, Hiroki Okumura, Yuko Arioka, Itaru Kushima, Daisuke Mori, Tzuyao Lo, Gantsooj Otgonbayar, Hidekazu Kato, Yoshihiro Nawa, Hiroki Kimura, Branko Aleksic, Norio Ozaki

**Affiliations:** 1 Department of Psychiatry, Nagoya University Graduate School of Medicine, Nagoya, Japan; 2 Department of Hospital Pharmacy, Nagoya University Hospital, Nagoya, Japan; 3 Pathophysiology of Mental Disorders, Nagoya University Graduate School of Medicine, Nagoya, Japan; 4 Center for Advanced Medicine and Clinical Research, Nagoya University Hospital, Nagoya, Japan; 5 Medical Genomics Center, Nagoya University Hospital, Nagoya, Japan; 6 Brain and Mind Research Center, Nagoya University, Nagoya, Japan; 7 Institute for Glyco-core Research (iGCORE), Nagoya University, Nagoya, Japan

**Keywords:** Stem cells, Clinical genetics

## Abstract

Recent genetic studies have found common genomic risk variants among psychiatric disorders, strongly suggesting the overlaps in their molecular and cellular mechanism. Our research group identified the variant in *ASTN2* as one of the candidate risk factors across these psychiatric disorders by whole-genome copy number variation analysis. However, the alterations in the human neuronal cells resulting from *ASTN2* variants identified in patients remain unknown. To address this, we used patient-derived and genome-edited iPS cells with *ASTN2* deletion; cells were further differentiated into neuronal cells. A comprehensive gene expression analysis using genome-edited iPS cells with variants on both alleles revealed that the expression level of *ZNF558*, a gene specifically expressed in human forebrain neural progenitor cells, was greatly reduced in *ASTN2*-deleted neuronal cells. Furthermore, the expression of the mitophagy-related gene *SPATA18*, which is repressed by *ZNF558*, and mitophagy activity were increased in *ASTN2*-deleted neuronal cells. These phenotypes were also detected in neuronal cells differentiated from patient-derived iPS cells with heterozygous *ASTN2* deletion. Our results suggest that *ASTN2* deletion is related to the common pathogenic mechanism of psychiatric disorders by regulating mitophagy via *ZNF558*.

## Introduction

Schizophrenia (SCZ), autism spectrum disorder (ASD), and bipolar disorder (BP) are very common psychiatric disorders, with high lifetime prevalence rates of ~0.5% [1, 2], 2.5% [3–5], and 1% [6, 7], respectively. They are characterized by their duration as chronic diseases, from childhood to the time of death. This results in a longer treatment period and extensive social and personal damage [8, 9]. However, the biological pathogenesis of these psychiatric disorders is not well understood. Their understanding is urgently needed to develop novel therapies that significantly improve the functional outcomes of patients.

The Diagnostic and Statistical Manual of Mental Disorders, Fifth Edition, has established the diagnostic criteria for SCZ, ASD, and BP as independent and separate diseases. In contrast, in clinical settings, the comorbid diagnoses are also allowed, and in some patients, drawing the diagnose line is difficult, suggesting that these psychiatric disorders partially share clinical symptoms and phenotypes. Indeed, studies reported that psychiatric disorders show common alterations, such as dysfunction in the white matter microstructure in the body of the corpus callosum [10] and brain responses in the ventral striatum during reward anticipation [11]. Additionally, recent genetic studies have uncovered common risk genomic variants in these psychiatric disorders [12–15]. These findings imply that there are common underlying mechanisms in these psychiatric disorders, prompting us to examine the molecular and cellular alterations caused by the common risk variants across these psychiatric disorders.

**Fig. 1:**
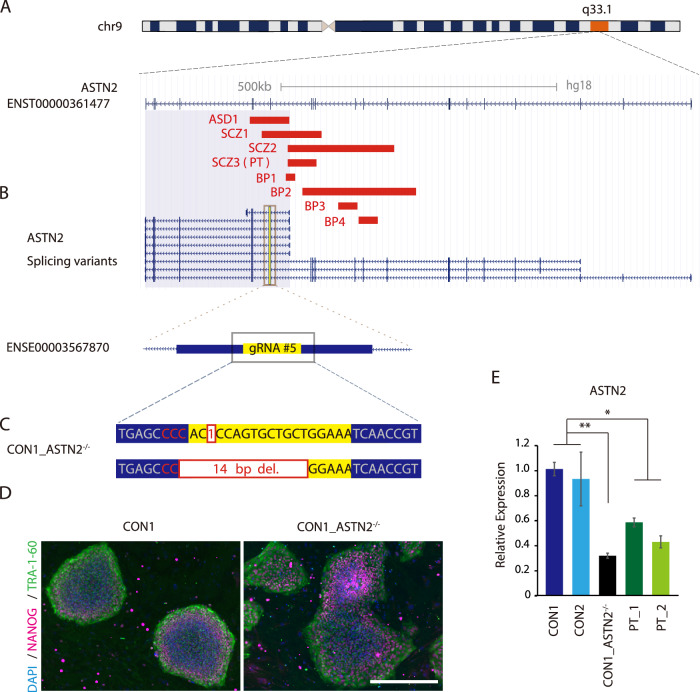
Establishment of *ASTN2*-deleted iPS cells using the CRISPR/Cas9 system. **A** Diagram of the deleted region of *ASTN2* in patients with psychiatric disorders identified by our previous CNV analysis. Red bars show the deletion region in each patient. **B** Diagram of *ASTN2* splicing variants. Exons in yellow are the most upstream exons of those common to all splicing variants. Regions in gray are those that have been previously identified as significantly associated with neurodevelopmental disorders. **C** The indel pattern of nonhomologous end joining. The red letters represent PAM sequences. **D** Immunostaining of pluripotent stem cell markers (NANOG and TRA-1-60) in iPS cell lines. The scale bar represents 500 µm. **E** RT-qPCR analysis of *ASTN2* in each iPS cell line. *N* = 3. Bars represent the mean ± standard error. Multiple comparison was performed using Dunnett’s test. *p < 0.05; **p < 0.01.

Recently, we identified the variants in *ASTN2* as a candidate risk factor for psychiatric disorders by whole-genome copy number variation (CNV) analysis [13, 16]. Moreover, a study reported that deletions in *ASTN2* that would disrupt almost all transcript isoforms (Fig. 1A, B, shaded gray region) are significantly enriched in patients with neurodevelopmental disorders, such as ASD, attention-deficit/hyperactivity disorder, and intellectual disability [17]. In support of these results, *ASTN2* is also listed in multiple databases, such as SFARI (https://gene.sfari.org/) and DBDGD (https://dbd.geisingeradmi.org/), which are centered on genes implicated in these psychiatric disorders.

*ASTN2* encodes a large vertebrate-specific transmembrane protein, astrotactin-2, which has been implicated in mouse models to regulate neuronal migration [18] and synaptic function [19]. In addition, *ASTN2* knockout mice show monoaminergic dysfunction and abnormal neuronal morphogenesis with shrinkage of the neuronal soma, implying some behavioral abnormalities and some emotional or cognitive impairments [20]. However, the molecular and cellular mechanisms caused by *ASTN2* variants identified in patients remain unknown. Moreover, considering the differences in structures and gene expressions between rodent and human brains, the examination of human neuronal cells is also required to understand the molecular and cellular alterations [21–23].

To address this, we applied a strategy using patient-derived and genome-edited iPS cells with *ASTN2* deletion. iPS cells have the genetic and molecular characteristics of individual subjects, allowing the researchers to overcome the limitations of animal models and to examine disease- and genetic-related phenotypes at the human cellular level [24, 25]. Here, we differentiated each iPS cell into neuronal cells and further examined them at the cellular and molecular levels. Our results suggest that as a pathogenic mechanism for psychiatric disorders, *ASTN2* deletion promotes mitophagy by downregulating *ZNF558* expression in human neuronal cells.

## Material and methods

### Subject

Previously, we had established iPS cells from one male Japanese patient with SCZ from eight patients with *ASTN2* deletion (SCZ3 in Fig. 1A, chr9:118,490,453–118,538,170, NCBI36/hg18) [26]. The two cloned iPS cell lines from this patient with SCZ were used in this study (PT_1 and PT_2). Two healthy Japanese subjects, a 34-year-old man (CON1) and a 30-year-old woman (CON2), were selected as the sources for control iPS cells, which had no pathogenic CNVs [27]. The given ages of the subjects were those at the time of the blood sampling for iPS cell generation. The use of human samples and genomic analyses were approved by the Ethics Committee of Nagoya University (approval number: 2012-0184). All subjects provided written informed consent.

### Establishment of iPS cells and differentiation into neurospheres (NSs)

The iPS cells were established from the peripheral blood of each subject and cultured as previously reported [26]. The cells were differentiated into NSs, as previously reported, with minor modifications [28]. After the passage of iPS cells onto feeder cells (Day 0), the iPS cells were cultured in an iPS cell medium comprising Dulbecco’s Modified Eagle Medium (DMEM/F12) supplemented with 20% knockout serum replacement (KSR), 2 -mM l-glutamine, 0.1-mM nonessential amino acids, 0.1-mM 2-mercaptoethanol, 100-units/mL penicillin, 100-μg/mL streptomycin, and 4-ng/mL basic fibroblast growth factor (bFGF). On day 1, SB431542 (3 μM), CHIR99021 (3 μM), and dorsomorphin (3 μM) were added to the iPS cell medium, and the iPS cells were incubated for 6 days (days 1–7). On day 7, they were dissociated into single cells with 0.5× TrypLE select and cultured in NS medium consisting of DMEM/F12 supplemented with 1× N-2 supplement, 0.6% glucose, 100-units/mL penicillin, 100-μg/mL streptomycin, 5-mM HEPES, 1× B-27 supplement, 20-ng/mL bFGF, 10-ng/mL hLIF, 10-μM Y-27632, 3-μM CHIR99021, and 2-μM SB431542 for 2 weeks (days 7–21). The cells were dissociated into single cells once on day 14 for passage. Then, the cells were cultured in a 5% CO_2_/18–22% O_2_ atmosphere during all experiments.

### Assessment of the size of NSs

The differentiated NSs (day 21) were imaged in a bright field using a BZ-X810 fluorescence microscope (KEYENCE, Japan). Induction into the NSs was performed twice independently, and 50 NSs were randomly selected for measurement. Each NS was identified as a circle or oval on the image using Adobe Illustrator 26.5 (Adobe Systems Inc, CA, USA), and subsequently, the area of each NS was measured using ImageJ.

### Evaluation of neurite outgrowth

On day 21, the differentiated NSs were dissociated into single cells and plated onto poly-l-ornithine/laminin/fibronectin-coated dishes in a pan-neuron medium (DMEM/F12 supplemented with 1× N-2 supplement, 0.6% glucose, 100-units/mL penicillin, 100-μg/mL streptomycin, 5-mM HEPES, 1× B-27 supplement, 10-μM DAPT, 20-ng/mL BDNF, 20-ng/mL GDNF, 0.2-mM ascorbic acid, 1-ng/mL TGF-β3, and 0.5-mM dbcAMP) for 3 days. For the mitophagy induction study, H_2_O_2_ (5 μM/20 μM) or rapamycin (0.5 μM/2 μM) was added as a mitophagy inducer [29, 30] and for the mitophagy inhibition study, chloroquine (0.1 μM/0.5 μM) was added as a mitophagy inhibitor to the pan-neuron medium. Timelapse images were acquired by IncuCyte ZOOM™ software (sartorius, MI, USA) for 3 days.

### Immunocytochemistry

The cultured cells were fixed with 4% paraformaldehyde for 15 min at room temperature, permeabilized and blocked in phosphate-buffered saline (PBS) containing 0.3% Triton X-100 and 1% bovine serum albumin for 60 min at room temperature, and then incubated with each of the primary antibodies overnight at 4 °C. After washing with PBS, the immunolabeled cells were incubated with appropriate fluorophore-conjugated secondary antibodies for 1 h at room temperature. DAPI (Dojindo, Japan) was used for nuclei. The antibodies used for immunocytochemistry are listed in Supplementary Table 1. Images were captured using a BZ-X810 fluorescence microscope.

Five images in each cell line were taken, and the number of βIII-tubulin positive, vGLUT1-positive, and DAPI-positive were counted. In measuring the neurite length, the 15 longest neurites were selected for each image. Neurite lengths were measured and calculated using ImageJ and its plug-in NeuronJ.

### Comprehensive expression analysis

Expression analysis was performed using the SurePrint G3 Human GE 8x60K V2 Microarray Kit (Agilent Technology, CA, USA), according to the manufacturer’s instructions. The sample number for the microarray was *n* = 3. All data (GEO: GSE260713) analyses were performed using the GeneSpring GX Software Program (version 13; Agilent Technology). Absolute expression values were normalized using quantile normalization. The statistical criteria for differential expression were as follows: moderated *t*-test, Bonferroni correction for multiple testing correction, corrected *p* value <0.05, and fold changes >2. Gene Ontology (GO) analysis (using GOTERM_BP_FAT, GOTERM_MF_FAT, and GOTERM_CC_FAT) was performed using DAVID’s functional annotation tool (https://david.ncifcrf.gov/).

### Analysis of *ZNF558* binding sites

To analyze the *ZNF558* binding sites to DNA, we used three sets of chromatin immunoprecipitation sequencing (ChIP-seq) data available on the Sequence Read Archive (SRA) (https://www.ncbi.nlm.nih.gov/sra) (SRR5197178, SRR5111604, SRR5111605). *ZNF224* (SRR5197084) was used as a control gene. The data were analyzed based on a previous report [31]. Briefly, adapter sequences were trimmed from raw datasets using Trimmomatic, followed by quality control using Fastq. Then, the data were mapped to the GRCh38 reference sequence using Bowtie2 with the sensitive-local setting. Multi-mapped reads were filtered out. Polymerase chain reaction (PCR) duplicates were then removed using samtools and repeat regions on the genome were removed using bedtools. Peak calling was performed using MACS2. Then, the processed called peaks were analyzed using GREAT, version 4.0.4 [32]. Binding sequence motif analysis was performed using HOMER.

### Evaluation of mitophagy

On day 21, NSs were dissociated into single cells and stained with Mtphagy Dye (Dojindo 1000:1) for 30 min in a 5% CO_2_/18–22% O_2_ atmosphere. The cells were washed with PBS and incubated in 96-well U-bottom plates at 5.0 × 10^4^ cells per well. Twenty-four hours later, they were stained with DAPI (Dojindo 5000:1) for 15 min, then dissociated again into single cells, and applied into BD FACS Canto II (BD Bioscience). The data were analyzed using FlowJo.

### Quantitative reverse transcription PCR (RT-qPCR) and measurement of mitochondrial DNA and VNTR (variable number tandem repeat) copy numbers

Total RNA was extracted using the RNeasy Plus Mini Kit (QIAGEN, Netherlands). Then, cDNA was generated using the High-Capacity RNA-to-cDNA Kit (Applied Biosystems, CA, USA). RT-qPCR was performed using KAPA SYBR Fast qPCR Kit (KAPA BIOSYSTEMS,) and detected using QuantStudio5 (Applied Biosystems). The gene expression values were normalized to the housekeeping gene RPS18. The primers for mitochondrial DNA (mtDNA) and nuclear DNA (nucDNA) copy number measurement were used as previously reported [33]. As previously reported, the VNTR copy numbers were measured [31]. Albumin was used as the positive control, and the values were calculated using the copy number of albumin as 2. The primers used are listed in Supplementary Table 2.

### Statistics

Statistical comparisons were conducted using repeated-measure multiple analysis of variance for the timelapse imaging study. For the other experiments, an unpaired *t*-test (two tails) or two-way analysis of variance followed by Dunnett’s post hoc test was used. The sample size was determined based on pilot data from our laboratory and previous similar studies. Statistical Package for the Social Sciences, version 28.0.1.0 (IBM, USA), was used to analyze the data. The significance level was set at 0.05 or 0.01.

## Results

### Deleted region of *ASTN2* in each patient with psychiatric disorders and establishment of *ASTN2*-deleted iPS cells by CRISPR/Cas9 genome editing

Our research group recently identified heterozygous *ASTN2* deletions in eight patients with psychiatric disorders (three patients with SCZ, one patient with ASD, and four patients with BP) by a whole-genome CNV analysis [13, 16, 34] (Fig. 1A). Among these patients, we could establish iPS cells from one patient (SCZ3), which were used in the subsequent analysis as PT cell lines in this study.

To understand the biological effect of *ASTN2* deletion on human neuronal cells, we also worked on the generation of an *ASTN2*-deleted iPS cell line from healthy control iPS cells (CON1) using the CRISPR/Cas9 system (Supplementary Methods). Five individual single-guide RNAs (sgRNAs) were constructed near the start point of the most upstream exon that all splicing variants have in common (ENSE00003567870, Fig. 1B and Supplementary Fig. 1A). Among the constructed sgRNAs, sgRNA#5 showed the strongest cleavage activity as examined by T7EI assay (Supplementary Fig. 1B). We also performed an off-target search using CCTop. CCTop predicted 13 potential off-target sites for sgRNA#5 in the human genome under the following conditions: core length = 12; max core mismatches = 2; max total mismatches = 3. However, the only target at an exonic position was in the *ASTN2* gene region (Supplementary Table 4). Taken together, sgRNA#5 was selected for *ASTN2*-targeted genome editing.

As a result, one *ASTN2*-deleted iPS cell line was established (CON1_ASTN2^−/−^ cell line). The deletion of each homologous chromosome in the CON1_ASTN2^−/−^ cell line was confirmed by Sanger sequencing, with a 1-bp deletion on one allele and a 14-bp deletion on the other (Fig. 1C). The CON1_ASTN2^−/−^ cell line expressed the pluripotent marker (TRA-1-60 and NANOG) as well as its parent iPS cell line CON1 (Fig. 1D), whereas the expression of *ASTN*2 in the CON1_ASTN2^−/−^ iPS cell line was reduced compared with that in CON1 (Fig. 1E). We also verified the downregulation of *ASTN2* expression in the PT_1 and PT_2 cell lines (Fig. 1E).

### Differentiation of iPS cells into NSs and measurement of expression levels of neuronal markers

**Fig. 2:**
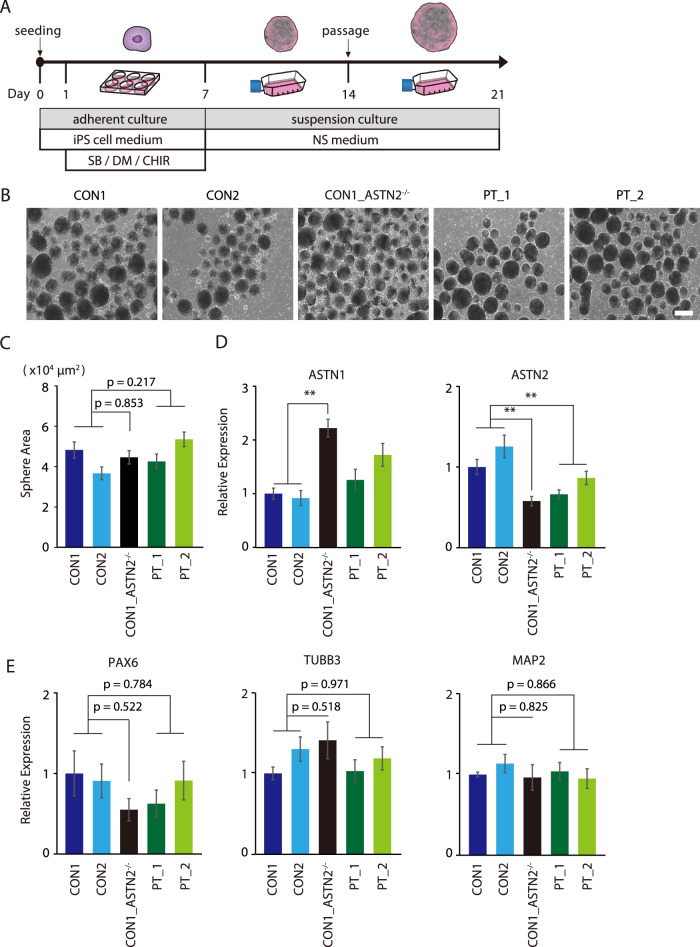
Differentiation of iPS cell lines into NSs. **A** Schematic timeline for NS differentiation from iPS cells. **B** Phage images of the NS (Day 21) of each cell line. The scale bar indicates 500 µm. **C** Comparison of NS area in each cell line. *N* = 50. Bars represent the mean ± standard error. Multiple comparison was performed using Dunnett’s test. **D**, **E** Relative mRNA expression levels for *ASTN1*, *ASTN2*, *PAX6*, *TUBB3*, and *MAP2* in NSs (day 21). *N* = 6. Bars represent the mean ± standard error. Multiple comparison was performed using Dunnett’s test. **p < 0.01.

To investigate the mechanisms of *ASTN2* deletion in neuronal cells, we differentiated each iPS cell line into NSs, which predominantly contain neuronal progenitors or stem cells. A differentiation scheme to NSs is shown in Fig. 2A. Each iPS cell line formed cell aggregates, including NSs, and no significant differences in their size were observed (Fig. 2B, C). Then, we examined *ASTN2* mRNA expression levels in the NSs on day 21. Compared with the parental cell lines, the CON1_ ASTN2^−/−^ and PT cell lines showed decreased mRNA expression of *ASTN2*, whereas the mRNA expression levels of *ASTN1* were significantly increased (Fig. 2D). No significant differences in the expression levels of *PAX6*, *TUBB3*, and *MAP2*, which are markers of the pan-neuron cells, were found between each cell line (Fig. 2E), suggesting that they are equally differentiated into the neuronal cells.

We also evaluated the expression of ASTN2 protein in neurospheres using immunoblotting using anti-ASTN2 antibody (Sigma-Aldrich #ZRB1283). We obtained a band at ~105 kDa that are expected size by the manufacturer’s document. However, the immunogen used to generate the anti-ASTN2 antibody (18 amino acids from the C-terminal half, lumenal domain of ASTN2) contains the short isoform region of ASTN2 (~50 kDa). Therefore, we evaluated the ASTN2 expression levels considering both the bands (~105 and ~50 kDa). The expression level of ~50 kDa ASTN2 rather than ~105 kDa was decreased in *ASTN2*-deleted neurospheres (patient-derived and genome-edited neurospheres) compared to control neurospheres (Supplementary Fig. 2).

### NSs derived from a patient with *ASTN2*-deleted SCZ and CON1_ASTN2^−/−^ cell line showed suppressed neurite outgrowth

Studies have shown that the decreased expression of psychiatric disorder-associated genes suppresses neurite outgrowth [27, 35–37]. Therefore, we observed how *ASTN2* deletion affects neurite outgrowth.

**Fig. 3:**
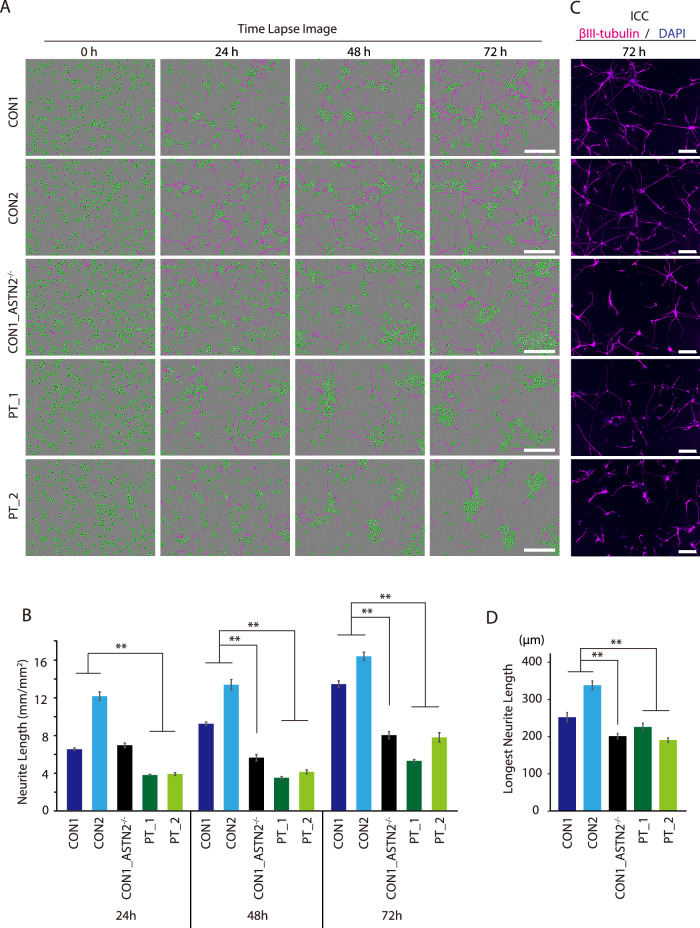
Assessment of neurite outgrowth in pan-neuronal cells derived from NSs by timelapse imaging and immunocytochemistry (ICC). **A** Timelapse images of pan-neuronal cell outgrowth of each cell line using IncuCyte ZOOM™ software. Scale bars indicate 200 µm. Green: cell body, Pink: neurite. **B** Neurite length measured in the indicated time after plating. *N* = 5. Bars represent the mean ± standard error. Repeated-measure multiple ANOVA method was used for analysis. Moreover, Bonferroni adjustment for multiple comparisons was performed. **C** Representative ICC images of pan-neuronal cells 72 h after plating. Immunostained for βIII-tubulin and DAPI. The white bars in the image indicate 200 µm. **D** Measurement of the longest neurite using ICC images. *N* = 75. Bars represent the mean ± standard error. Multiple comparison was performed using Dunnett’s test. **p < 0.01.

NSs from each cell line were dissociated into single cells once and seeded onto poly-L-ornithine/laminin/fibronectin-coated plates and observed by timelapse imaging for 3 days. As a result, the CON1_ASTN2^−/−^ and PT cell lines showed suppressed neurite outgrowth compared with the control cell lines at almost all time points (Fig. 3A, B). In agreement with this, the immunocytochemistry analysis showed that the βIII-tubulin (a marker for neurons)-expressing neurites in *ASTN2*-deleted cell lines were shorter than those in the control cell lines 72 h after seeding onto the plates (Fig. 3C, D). Considering that no differences in the ratio of βIII-tubulin- and vGLUT1-expressing cells were observed between cell lines (Supplementary Fig. 3), the suppressed neurite outgrowth in *ASTN2*-deleted cell lines was not due to the differences in the degree of differentiation of each cell line.

### Comprehensive expression analysis of the CON1_ ASTN2^−/−^ cell line compared with its parental cell line showed decreased expression of *ZNF558*

**Fig. 4:**
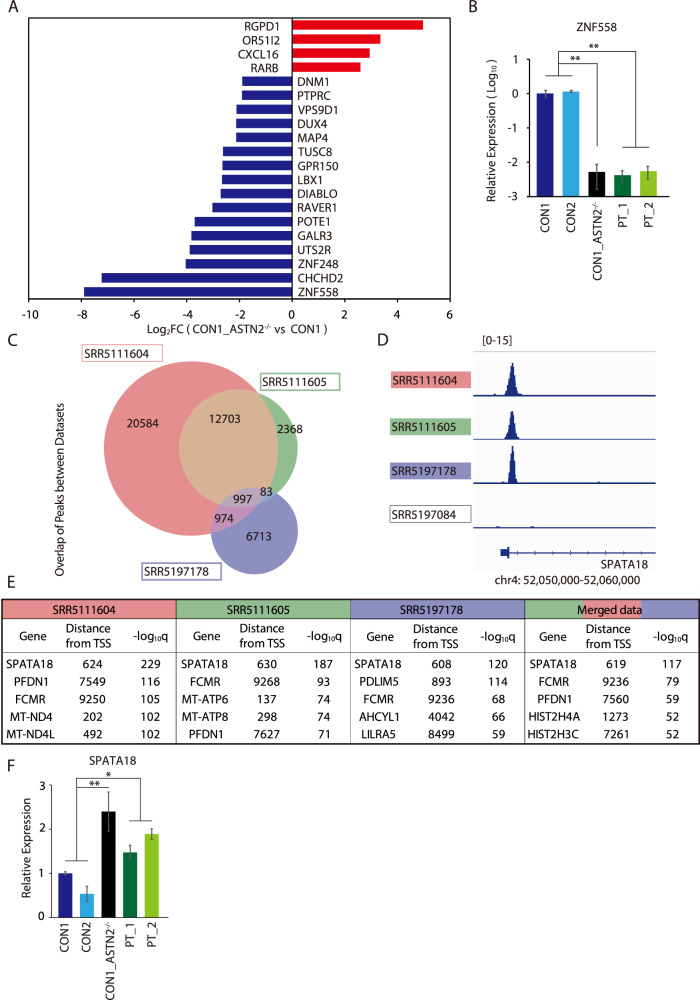
Comprehensive gene expression and ChIP-seq analyses identified *ZNF558* and *SPATA18.* **A** Differentially expressed genes (protein coding) with fold change >2, Bonferroni correction of *p* value <0.05. Red/blue indicates genes that have increased/decreased in CON1_ASTN2^−*/−*^ *vs*. CON1. **B** Relative mRNA expression level of *ZNF558*. *N* = 6. Bars represent the mean ± standard error. Multiple comparison was performed using Dunnett’s test. **p < 0.01. **C** Number of peaks overlapping between datasets. **D** Image of called peaks most plausible in each dataset. **E** Five most significant genes around the peaks called for each ChIP-seq dataset and its distance from TSS. **F** Relative mRNA expression level of *SPATA18*. *N* = 6. Bars represent the mean ± standard error. Multiple comparison was performed using Dunnett’s test. *p < 0.05; **p < 0.01.

Next, to explore the molecular and biological effects of *ASTN2* deletion, CON1_ASTN2^−/−^ and its parental NSs were applied to the comprehensive gene expression analysis. The analysis of differentially expressed genes in the CON1_ASTN2^−/−^ cell line identified 1241 upregulated entities and 741 downregulated entities (*T*-test cutoff *p* < 0.05, fold change >2, Supplementary Fig. 4A, and Supplementary Table 5). The upregulated genes were mainly associated with cell localization or motility (Supplementary Fig. 4B and Supplementary Table 6) In contrast, the downregulated genes were enriched in GO terms in transcription factor activity (Supplementary Fig. 4B and Supplementary Tables 6,7). The Bonferroni correction for multiple comparisons corrected 1,982 entities to 42 entities (including four upregulated and 16 downregulated protein-coding genes), of which *ZNF558* had been identified as the most downregulated gene in the CON1_ASTN2^−/−^ cell line compared with the parental NSs (Fig. 4A). The expression of *ZNF558* was extremely downregulated in the subsequent RT-qPCR in both the CON1_ASTN2^−/−^ and PT cell lines (Fig. 4B). This result suggests that *ASTN2* deletion is involved in the pathogenesis of psychiatric disorders by downregulating the expression of *ZNF558*. Therefore, we focused on *ZNF558* and performed further analysis.

### Analysis of the DNA binding sites of *ZNF558*

The zinc finger protein 558 encoded by *ZNF558* is a protein that belongs to the Krüppel-associated box domain zinc finger protein (KZFP) family and is known to bind to DNA [38, 39]. A report suggested that *ZNF558* binds with the DNA region of *SPATA18*, a gene involved in mitophagy [31]. We attempted to confirm the reproducibility by analyzing multiple ChIP-seq datasets targeting *ZNF558* from public databases.

We used three sets of ChIP-seq data available on the SRA (https://www.ncbi.nlm.nih.gov/sra) (SRR5197178, SRR5111604, and SRR5111605) for *ZNF558* using the HEK293 cell line. SRR5111604 and SRR5111605 were duplicate data from the same sample (Fig. 4C). The analysis pipeline and the result of region-gene association is shown in Supplementary Fig. 5A–D. The highest scoring peak was called near the transcriptional start sight of *SPATA18* in each dataset and even merged data from the three datasets (Fig. 4D, E and Supplementary Table 8). In agreement with this result, RT-qPCR analysis showed that the expression level of *SPATA18* was significantly increased in the PT and CON1_ASTN2^−/−^ cell lines compared with that in the control cell lines (Fig. 4F).

### Increased mitophagy and decreased ratio of the copy number of mtDNA/nucDNA in the CON1_ ASTN2^−/−^ and PT cell lines

**Fig. 5:**
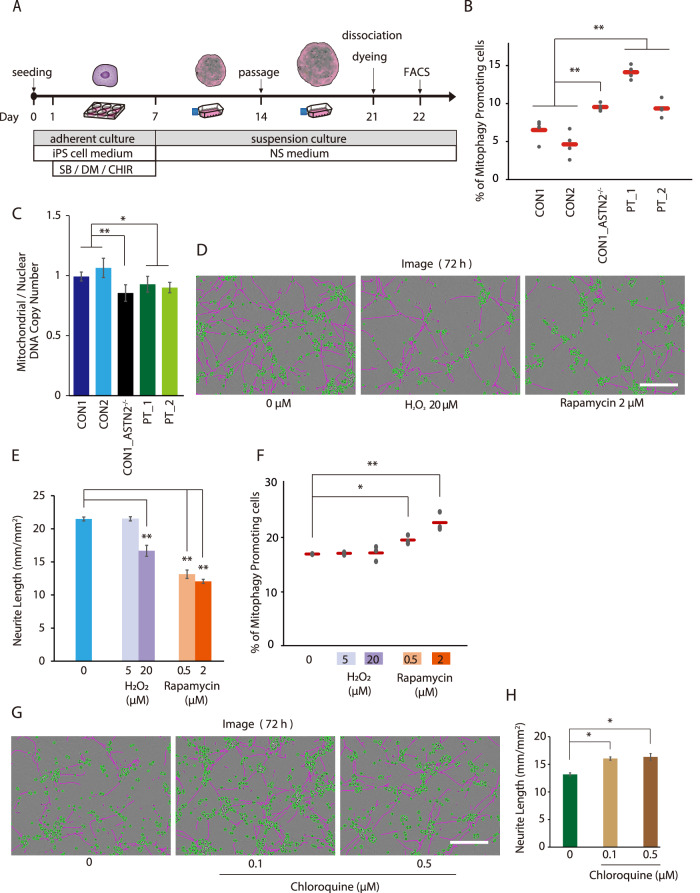
Analysis of mitophagy in NSs. **A** Schematic timeline for flow cytometry analysis. **B** Results of flow cytometry analysis. *N* = 4. The red bar indicates the average value. Multiple comparison was performed using Dunnett’s test. **p < 0.01. **C** MtDNA/nucDNA copy number ratio. *N* = 6. Bars represent the mean ± standard error. Multiple comparison was performed using Dunnett’s test. *p < 0.05; **p < 0.01. **D** Representative images of the CON2 cell line with H_2_O_2_ or rapamycin. Images were obtained 72 h after plating. Green: cell body, Pink: neurite. The scale bar indicates 200 µm. **E** Neurite length of the CON2 cell lines 72 h after H_2_O_2_ or rapamycin was added. *N* = 4. Bars represent the mean ± standard error. Multiple comparison was performed using Dunnett’s test. **p < 0.01. **F** The results of the flow cytometry analysis with the addition of H_2_O_2_ or rapamycin. *N* = 3. Multiple comparison was performed using Dunnett’s test. *p < 0.05; **p < 0.01. **G** The representative images of the PT cell line with 0.1 µM or 0.5 µM chloroquine. The images were obtained 72 h after plating. Green, cell body; pink, neurite. The scale bar indicates 200 µm. **H** The neurite length of the PT cell line was 72 h after 0.1 µM or 0.5 µM chloroquine was added (*N* = 4). The bars represent the mean ± standard error. Multiple comparison was performed using Dunnett’s test. *p < 0.05.

According to previous reports, the mitophagy-related gene *SPATA18* (also known as mitochondria-eating protein) enhances mitophagy in the extracellular matrix [31, 33, 40]. Therefore, we compared the intensity of mitophagy among the cell lines. The NSs were stained with mitophagy dye on day 21, and their intensity was measured using flow cytometry. The experimental scheme is shown in Fig. 5A. As a result, the PT and CON1_ASTN2^-/-^ cell lines increased the fraction of high fluorescence intensity by 2–3 folds compared with the control cell lines (Fig. 5B and Supplementary Fig. 6A,B). Additionally, previous reports suggest that increased mitophagy consequently alters the mtDNA/nucDNA copy number ratio [31, 40]. In agreement with the result of flow cytometry analysis, we found that the mtDNA/nucDNA copy number ratio was reduced in the PT and CON1_ASTN2^−/−^ cell lines (Fig. 5C). These results confirm the enhanced mitophagy in *ASTN2*-deleted cell lines.

Finally, we investigated whether the increased mitophagy affects neurite outgrowth. As shown in Fig. 5D–F, H_2_O_2_ [30] and rapamycin [29], which increase mitophagy, attenuated the neurite outgrowth of CON2 cell lines compared with the vehicle. Moreover, we found that chloroquine, an inhibitor of mitophagy, improved neurite outgrowth in the PT cell line (Fig. 5G, H). Overall, these findings suggest that the impaired neurite outgrowth of ASTN-deleted cells is attributed to mitophagy promotion.

## Discussion

**Fig. 6:**
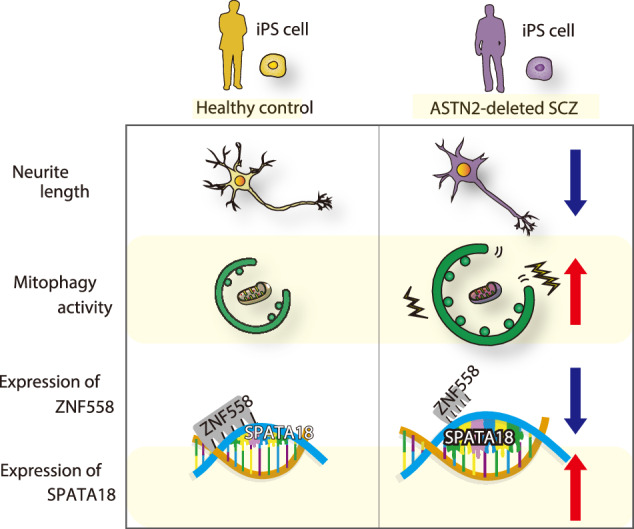
Graphical abstract of this research.

In this study, we showed how *ASTN2* deletion, a candidate cross-disorder risk variant for psychiatric disorders, affects human neuronal cells. To the best of our knowledge, this study is the first report of molecular biological analysis using patient-derived iPS cells with *ASTN2* deletion. We found that both the patient-derived and genome-edited *ASTN2*-deleted neuronal cells showed shortened neurite outgrowth, reduced expression of *ZNF558*, increased expression of *SPATA18*, increased mitophagy, and reduced mtDNA/nucDNA copy number ratio compared with healthy control neuronal cells (Fig. 6). These findings suggest that *ASTN2* is involved in regulating mitochondrial quality, which may be implicated in the pathogenesis of various psychiatric disorders.

To determine the effects of *ASTN2* deletion in human neuronal cells, we performed gene editing, in addition to using iPS cells derived from a patient with *ASTN2* deletion. The exons we targeted using the CRISPR/Cas9 system to create *ASTN2*-deleted cell lines were not the same as the deleted exons in the SCZ3 patient. The CON1_ASTN2^−/−^ and PT cell lines differed in the following aspects: frameshift due to single and 14 bases of deletion in ENST00000313400.9 exon 17, or a frameshift due to 410 bases of deletion over exons 14, 15, and 16. However, these two cell lines showed almost similar phenotypes, in terms of the degree of neurite outgrowth, the mRNA expression of *ZNF558* and *SPATA18*, and the intensity of mitophagy. To address this, we aimed to quantify the ASTN2 protein of neurospheres. *ASTN2*-deleted neurospheres (patient-derived and genome-edited neurospheres), showed a decrease in ~50 kDa ASTN2 protein levels (Supplementary Fig. 2). *ASTN2* has many splicing variants (Fig. 1B). Thus, it is probable that the ~50 kDa ASTN2 protein is the mainly expressed variant in neurospheres and the ~105 kDa band protein is a nonspecific one. However, the unexpected result in which the genome-edited cells (CON1_ASTN2^−/−^)—with biallelic out-of-frame variants leading to an almost complete knockout of *ASTN2*—continued to express ASTN2 protein, remains unexplained. The potential limitations of the CRISPR technology may explain this result. Genome editing using the CRISPR-Cas9 system may sometimes cause the expression of unanticipated proteins, similar in size to the wild-type protein, from the out-of-frame variant alleles [41, 42]. Moreover, the non-sense mediated mRNA decay efficiency of CRISPR-Cas9 system-induced frameshift knock-out variants is inconsistent; and complete loss of protein expression is not achieved [43]. This may explain the continued expression of ASTN2 in our genome-edited cells (CON1_ASTN2^−/−^ cells).

Based on our results, it seems that patient-derived and genome-edited cells have the similar reduced expression level of ASTN2. Hence, it can be concluded that these phenotypes are caused by the reduced expression of *ASTN2*. Moreover, given that this region of *ASTN2*, including the target site of CRISPR/Cas9 and the deleted region in SCZ3, is a disease-susceptible region (gray-shaded area in Fig. 1A) [17], these phenotypes will also reflect the vulnerabilities for the onset of psychiatric disorders at the cellular and molecular levels.

The brain is composed of various types of neuronal and glial cells, all of which are generated by the differentiation of neural stem cells (NSCs). Such NSC activities from embryonic to early childhood must be precisely controlled. The function of transcription factors, a protein family that regulates gene transcription, is crucial for differentiating NSCs into various nervous system cells and their subsequent formation into various brain regions [44]. In a study of postmortem brains, Voineagu et al. revealed that gene expression dysregulation related to brain development in the embryonic period may be associated with the occurrence of ASD [45]. Furthermore, a recent CNV analysis study of patients with SCZ and ASD has shown that CNV was significantly enriched in enhancers and promoters in brain tissue, suggesting that gene expression dysregulation is involved in the pathogenesis of SCZ and ASD [13]. C2H2 zinc finger proteins are the largest family of human transcription factors [46] and are highly expressed in the developing brain. They have been shown to regulate transcription patterning of the early central nervous system and play an important role in regulating brain development [47]. A 335.4-kb duplication in the Xp11.2p11.3 region, which contains the C2H2 zinc finger proteins, *ZNF81* and *ZNF182*, was identified in a patient showing developmental delays, autistic features, and growth and speech delays [48]. Another study on a large cohort of probands with ASD in China identified *ZNF292* as a candidate risk gene [49]. Additionally, another analysis of the Autism Genetic Research Exchange cohort, consisting of approximately 1,000 multiplex ASD families, identified homozygous and compound heterozygous variants in the *ZNF18* gene [50]. The KRAB zinc finger protein family, a subtype of the C2H2-ZNF protein family characterized by its ZNF and KRAB domains, is expressed characteristically in the human brain compared with other primates, such as chimpanzees. The KRAB zinc finger protein is necessary for the transcriptional network that drives the unique features of the human brain, such as increased cognitive activity and larger brain size [47]. In this study, we found that the *ZNF558* gene was most differentially expressed in the CON1_ASTN2^−/−^ cell line compared with that in its parental cell line. This result was also confirmed in the PT cell line, a heterozygous cell line with *ASTN2* deletion. Another report has shown that not only by the knockout of *ASTN2* but also by the knockout of other ASD-related genes, such as *ATRX*, *AFF2*, *KCNQ2*, *SCN2A*, and *DLGAP2*, the expression of *ZNF558* is decreased [51]. Similarly, the expression of several other C2H2-ZNF family proteins was also decreased by the knockout of ASD-related genes [51]. These findings suggest that phenotypes detected in the ASTN2-deleted neurons, which are deficient in *ZNF558* expression, may reflect the dysregulation of transcriptional systems or dysfunction of some higher brain functions related to ASD and SCZ.

Another aspect of the important function of NSCs in normal brain development is neural cell migration [52]. The important thing is to migrate to the right place at the right time. Reports have shown that functional impairment of proteins involved in neural cell migration, such as *RELN* [53–55] and *NRG1*/*ERBB4* [56], is also associated with the pathogenesis of some neurological syndromes, including SCZ and BP [57]. *ASTN1* is a ligand for neuron–glia junctions during the migration of neural progenitors along the radial glia in early embryos. In contrast, rather than directly controlling neuron–glia adhesions, *ASTN2* regulates *ASTN1* surface expression by forming a complex with *ASTN1* in endocytosis—indirectly controlling the neural cell migration [58]. Thus, *ASTN1* and *ASTN2* complementarily and conjugately regulate neuronal migration during brain development. As an important case study, Mitani et al. reported brain malformation and abnormally shaped skulls as a result of the presence of double-heterozygous variants of both *ASTN1* and *ASTN2* [59]. These findings suggest that *ASTN2* and *ASTN1* play important roles in neuron localization and brain morphogenesis in early embryos or immature nervous systems. In this study, the mRNA expression level of *ASTN1* was increased in both the PT and CON1_ASTN2^−/−^ NSs compared with that in healthy control NSs. NSs consist of immature neural progenitors. Considering the indispensability and cooperativeness of *ASTN1* and *ASTN2*, the upregulation of *ASTN1* expression in *ASTN2*-deleted NSs may be due to compensation for the deletion of *ASTN2*. In fact, in the comprehensive expression analysis of NSs conducted in this study, the upregulated genes in the *ASTN2*-deleted cell line were most enriched in the category related to cell migration, cell localization, and cell motility.

In this study, we analyzed the DNA binding sites of *ZNF558* using ChIP-seq data on the SRA. These are the results performed using HEK293T cells; therefore, we cannot rule out the possibility of different results when performed using neuronal cells, such as NSs. Nevertheless, the results were considered reasonable, as all three datasets showed the most specific binding to the transcription start sites (TSS) flanking region of *SPATA18*, and mRNA expression levels in the NS also showed differences between cell lines. Furthermore, a report confirmed that knocking out *ZNF558* predominantly increased *SPATA18* expression [31], which is consistent with the present results. This report also indicated that the expression of *ZNF558* is regulated by the copy number of its downstream VNTR [31]. Therefore, we measured the respective VNTR copy numbers in the healthy control, PT, and CON1_ASTN2−/− cell lines and found that the VNTR copy number increased in the PT cell lines but did not differ from that in the healthy control in the CON1_ASTN2−/− cell lines (Supplementary Fig. 7). This suggests that a causal relationship was observed between *ASTN2* and *ZNF558* other than by VNTR copy number. As mentioned previously, the knockout of other ASD-related genes, aside from *ASTN2*, also causes the decreased expression of *ZNF558*. The identification of the regulatory system of *ZNF558* downregulation will cut through the revealing molecular mechanism of psychiatric disorders, which should be addressed in future research.

We confirmed increased levels of mitophagy and reduced mtDNA/nucDNA copy number ratio in the PT and CON1_ASTN2^−/−^ cell lines, both of which showed upregulated expression of *SPATA18*. According to a previous report, the overexpression of *SPATA18* may efficiently eliminate abnormal mitochondria and reduce the mtDNA/nucDNA ratio [60]. Abnormal mitochondrial morphology has been reported in various neurodevelopmental disorders, including SCZ and tuberous sclerosis, which is a comorbidity associated with ASD in approximately half of patients [61–63]. Additionally, reduced numbers and size of mitochondria have been observed in SCZ patients [64, 65]. It is possible that abnormal mitochondria accumulate in the PT and CON1_ASTN2^−/−^ cell lines where mitophagy eliminates these abnormal mitochondria.

We found that neurite outgrowth is inhibited in *ASTN2*-deleted neuronal cells and this impairment is associated with their increased mitophagy. Neurites develop immediately after a neuron differentiates from NSCs, and one of several neurites begins to elongate rapidly and takes on the characteristics of an axon during brain development. On that occasion, mitochondria aggregate at the base of the future axon before neurite outgrowth, indicating that mitochondria play an important role in neurite outgrowth [66]. Moreover, if treated with EtBr, mtDNA is selectively and irreversibly damaged, and cells are no longer able to gain polarity and develop axons [67]. Based on the findings of these previous reports and those of the present study, further research on mitochondria is needed to better understand the underlying mechanisms of psychiatric disorders based on *ASTN2* deletion.

Here, we used rapamycin and H_2_O_2_ to induce mitophagy. Rapamycin induces mitophagy by inhibiting the PINK1 and PRKN-activated mTOR pathway [68]. Indeed, rapamycin-treated cells showed increased mitophagy with shortened neurites (Fig. 5D–F). Chloroquine, an inhibitor of mitophagy, improves neurite outgrowth. Thus, the impaired neurite outgrowth in rapamycin-treated cells is probably due to increased mitophagy. In contrast, H_2_O_2_-treated cells show impaired neurite outgrowth, but mitophagy remains unaltered (Fig. 5D–F). H_2_O_2_, a reactive oxygen species (ROS), induces mitophagy by generating highly oxidative hydroxyl radicals [69]. Counterintuitively, H_2_O_2_ may inhibit autophagy by impairing LC3 lipidation which is a key process for autophagosome induction and autophagosome-lysosome integration [70]. This may explain why H_2_O_2_-treatment failed to increase mitophagy in this study. Moreover, cell damage induced by ROS may explain the shortened neurites observed in the H_2_O_2_-treated cells, here.

Our final goal was to elucidate the molecular and cellular pathophysiology in the brains of patients with *ASTN2* deletion. To address this, we used induced pluripotent stem cell-derived pan-neuronal cells as a lead to understand them in this study. Pan-neuronal cells mainly consist of cortical neurons but partially include the nonspecific neuronal subtype, and, therefore, are useful for getting an overview of the findings in neuronal cells. Indeed, *ASTN2*-deleted pan-neuronal cells provided us with novel findings, as mentioned above. For the next strategy, we can focus on each neuronal subtype, such as Purkinje cells in the cerebellum, where *ASTN2* is highly expressed. Large-scale de novo variant analyses in patients with ASD have shown that ASD-related candidate genes in early embryos are enriched in the cerebellum [71], and cerebellar dysfunction seems deeply implicated in the pathophysiology of ASD [72]. Moreover, Purkinje cells are representative cells with increasing rates of mitophagy [30]. Because the technology to generate cerebellar organoids from iPS cells has been recently reported, it will be possible to apply that technology to our iPS cells [73]. Starting with our study, more detailed analyses are expected to achieve the goal.

This study has several limitations. First, variants of *ASTN2* have been identified in several psychiatric disorders such as SCZ, ASD, and BP. However, we could only access the SCZ patient with *ASTN2* deletion to generate iPS cells, which is considered the main limitation of this study. To determine the mechanism of cross-disorder psychiatric disorders based on *ASTN2*, further studies using iPS cells derived from ASD and BP patients are needed. Second, validation of our findings by direct analysis of neurons in a fetal patient’s brain is not feasible because psychiatric disorders are not diagnosed during the fetal period. However, for over three decades, SCZ has been regarded as a neurodevelopmental disorder [74]. Consistent with this, patient iPS cell-derived neurons often exhibit shortened neurites [75, 76], implying that the impairment of neurite length during the neurodevelopmental stage is a hallmark of psychiatric disorders, including SCZ. Therefore, our findings will contribute to reveal the mechanism of psychiatric disorders in addition to previous studies.

In conclusion, by studying disease- and genetic-related phenotypes at the human cellular level, this study provided one possible hypothesis on how *ASTN2*, a candidate cross-disorder risk variant for psychiatric disorders, is involved in the pathogenesis of these disorders and throws new insights into the understanding of their molecular and cellular mechanisms. This research will play a role as a stepping stone to reveal the pathomechanisms of psychiatric disorders and contribute to the development of even more effective therapies in the future.

### Supplementary information

Supplementary Material

Supplementary table 5

Supplementary table 6

Supplementary table 7

Supplementary table 8

## Data Availability

All data supporting our findings can be found in the main paper or in supplementary files. Gene expression data are available via the GEO accession number: GSE260713.
