# Supplementary material for: Analysis of human neuronal cells carrying ASTN2 deletion associated with psychiatric disorders
Source: Transl Psychiatry. 2024 Jun 3;14:236. doi: 10.1038/s41398-024-02962-4 (PMC11148150; doi:10.1038/s41398-024-02962-4)
Supplement: Supplementary file 1 — Supplementary Material [file 41398_2024_2962_MOESM1_ESM.docx]

**Supplementary Methods**

**DNA constructs for clustered regularly interspaced short palindromic repeat (CRISPR)/CRISPR-associated 9 (Cas9) systems**

We generated each expression vector as in a previous study^1^. A Cas9 expression vector (pHL-EF1α-SphcCas9-iP) with a puromycin-resistant cassette was obtained from Addgene (ID: 60599). To construct sgRNA expression vectors, two oligos containing the sgRNA target site and a universal reverse primer (Supplementary Table 3) were amplified using polymerase chain reaction and cloned into the BamHI-EcoRI site of the pHL-H1-ccdB-mEF1α-RiH vector (Addgene ID: 60601) with a RFP cassette. All procedures were conducted according to the guidelines for recombinant DNA research established by Nagoya University.

**Transfection of CRISPR into human induced pluripotent stem cells (iPSCs)**

For the transfection of CRISPR into human iPSCs, feeder-free cultured CON1-derived iPSCs were pretreated with Y-27632 at 10 μM for at least 1 h and dissociated into single cells by treatment with TrypLe Select for 5 min at 37°C. A suspension containing 1 × 10^6^ cells was then transfected with Cas9 and sgRNA using FuGENE HD (Promega, USA). The cells were plated onto one Matrigel-coated well of a six-well plate under feeder-free conditions in the presence of 10-μM Y-27632 for 24 h. Forty-eight hours after transfection, 2-μg/mL puromycin was added for selection. Twenty-four hours later, the cells were cultured in mouse embryonic fibroblast-conditioned medium until they formed large colonies.

**T7 endonuclease I (T7EI) assay**

Puromycin-selected cells were used for the T7EI assay to examine sgRNA activity. The target region of sgRNAs was amplified from genomic DNA by a high-fidelity PCR using the primers shown in Supplementary Table 3. The PCR products were denatured (95°C for 2 min) and re-annealed (85°C–25°C at −0.1°C/s), followed by T7E1 (NEB) digestion for 30 min at 37°C. The digested products were analyzed on 1.5% agarose gels.

**Selection of genome-edited iPSCs**

The iPSCs were transfected with Cas9 and sgRNA#5 and allowed to grow into large colonies. Single iPSC colonies were picked from the dish using a pipetman under visual guidance (under a microscope) and transferred into individual wells of a 96-well plate containing iPSC medium. The transferred single large colony was pipetted up and down to break up to smaller clumps, and transferred into individual wells of a 24-well plate containing feeder cells and iPSC medium and were allowed to form large colonies. Subsequently, we extracted genomic DNA from colonies in each well and examined if they have mutations in the target *ASTN2* region using Sanger sequencing.

1. Li, H. L. *et al.* Precise Correction of the dystrophin gene in Duchenne muscular dystrophy patient induced pluripotent stem cells by TALEN and CRISPR-Cas9. *Stem Cell Reports* 2015; 4: 143–154.

**Supplementary Figures**


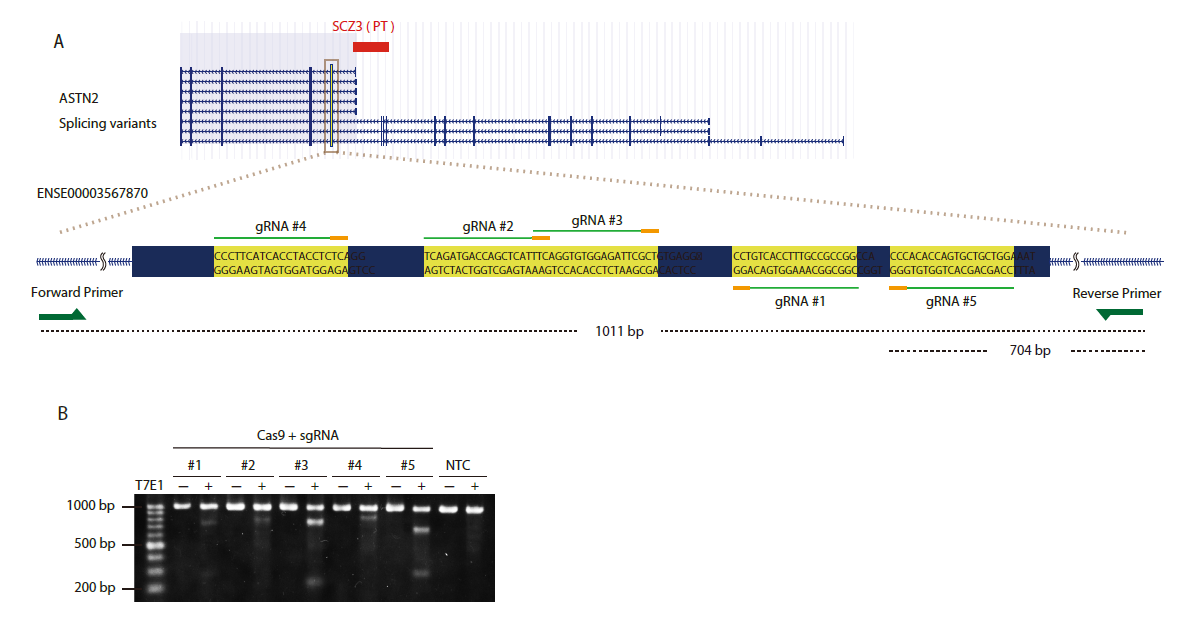


Supplementary Figure 1. Generation of the CON1_ASTN2*^-/-^* cell line using the CRISPR/Cas9 system.

(A) Diagram of sgRNA sequences. Orange bar indicates PAM sequences. The primer pair used in the T7E1 assay is shown using a green arrow. (B) Result of electrophoresis in the T7E1 assay.


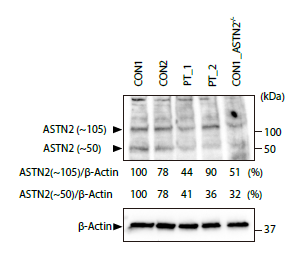


Supplementary Figure 2. Immunoblotting for ASTN2.
Immunoblotting for ASTN2 using proteins extracted from the neurospheres. The numbers indicate the representative relative values (CON1 = 100). The used antibodies were as follows: anti-ASTN2 antibody (Sigma-Aldrich #ZRB1283) and anti-β actin (Sigma-Aldrich #A3854).


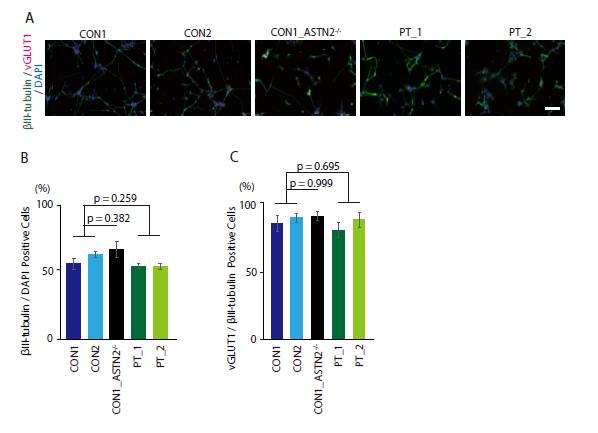
Supplementary Figure 3. Immunocytochemistry for βIII-tubulin and vGLUT1

(A) The representative images of neurons immunostained for βIII-tubulin and vGLUT1 72 h after plating. The white bars in the image indicate 200 µm. (B) The ratio of βⅢ-tubulin-positive cells. Bars represent mean ± standard error. Multiple comparison was performed using Dunnett’s test. Five images were obtained for each cell line. The total number of cells analyzed for each cell line is as follows: CON1, N = 472; CON2, N = 456; CON1_ASTN2*^-/-^*, N = 430; PT_1, N = 371; PT_2, N = 409. (C) The ratio of vGLUT1-positive cells. Five images were obtained for each cell line. The total number of cells analyzed for each cell line is as follows: CON1, N = 76; CON2, N = 78; CON1_ASTN2*^-/-^*, N = 55; PT_1, N = 64; and PT_2, N = 49. The bars represent the mean ± standard error. Multiple comparison was performed using Dunnett’s test.


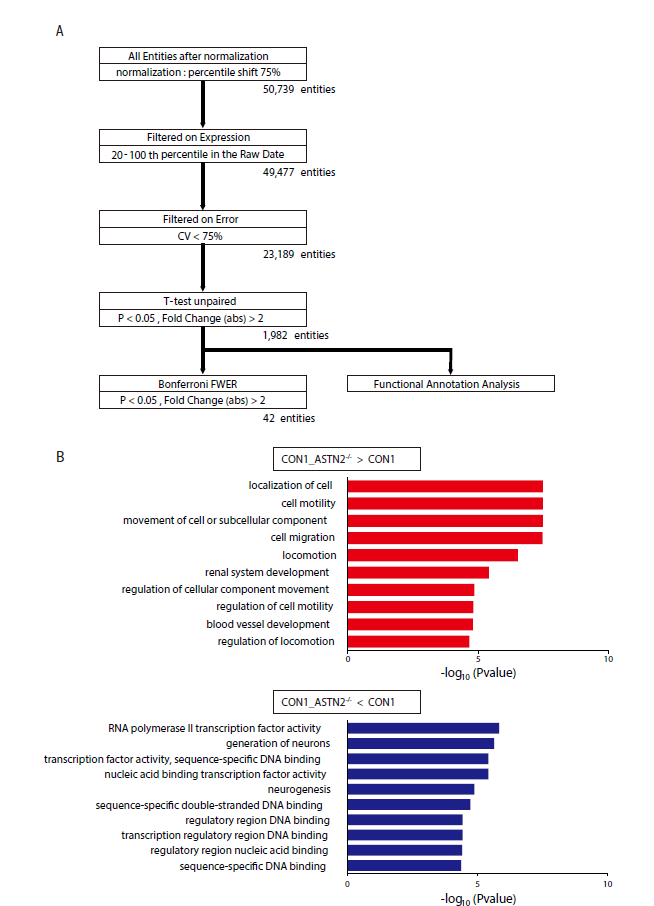


Supplementary Figure 4. Comprehensive gene expression and subsequent analyses.

(A) Diagram of the analysis pipeline. (B) GO analysis identified enriched categories for the altered genes in CON1_ASTN2*^-/-^* NSs. Annotation cluster list of the top 10 categories showing the smallest *p*-values.


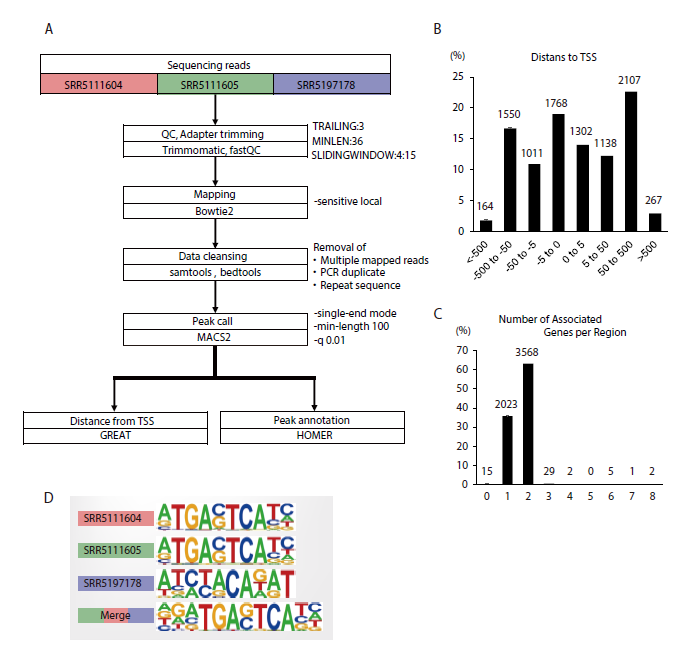


Supplementary Figure 5. Pipeline of ChIP-seq analysis and results of the analysis.

(A) Diagram of the analysis pipeline for the three ChIP-seq datasets available on the SRA. (B) Distance between input regions and their putatively regulated genes obtained from merged data. The number above each bar represents the number of genomic regions. (C) The number of associated genes assigned in each genomic region as putatively regulating. The number above each bar represents the number of genomic regions. (D) Results of *ZNF558* binding motif analysis for each dataset and merged data.


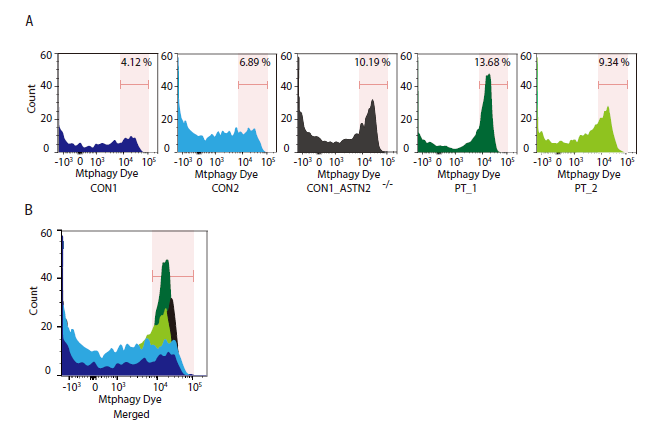


Supplementary Figure 6. Analysis of mitophagy in NSs using flow cytometry.

(A) Representative result of flow cytometry visualized by FlowJo software with a biexponential transformation. Fluorescence intensity of the mitophagy dye in each cell line. The population of cells emitting fluorescence intensities over the threshold shown in pink (= mitophagy-promoting cells). (B) Merged histogram of (A).


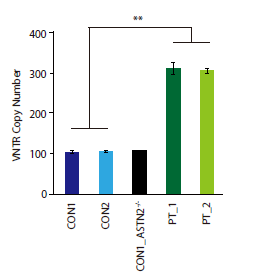

Supplementary Figure 7. VNTR analysis
The quantification of the copy number of VNTR using the DNA from the neurospheres (N = 3). The bars represent the mean ± standard error. Multiple comparison was performed using Dunnett’s test. ** *p* < 0.01.

**Supplementary Tables**

**Supplementary Table 1. Antibodies**

| antibodies | source | identifier |
| --- | --- | --- |
| Rabbit anti-NANOG, polyclonal | Abcam | Cat# ab21624 |
| Mouse anti-TRA-1-60, monoclonal | Abcam | Cat# ab16288 |
| Mouse anti-βIII-tubulin, monoclonal | Sigma | Cat# T8660 |
| Rabbit anti-VGLUT1, antiserum | Synaptic Systems | Cat# 135 302 |
| Donkey anti-mouse IgG (Alexa Fluor® 488) | Abcam | Cat# ab150109 |
| Donkey anti-rabbit IgG (Alexa Fluor® 594) | Abcam | Cat# ab150064 |

**Supplementary Table 2. Primer sequence for RT-qPCR**

| Targets | Primers | Sequence (5’→3’) |
| --- | --- | --- |
| *ASTN2* | ASTN2-Fw | ACCTACCTCTCAGGTTTGCT |
|  | ASTN2-Rv | ACCAGTAGGAACTCATCAGTG |
| *ASTN1* | ASTN1-Fw | GGTTCTCTTCTCTCAGCAGAACTC |
|  | ASTN1-Rv | AGGTGCTTATGTACTGGATCCTTC |
| *ZNF558* | ZNF558-Fw | CCAAGCACCTGTCCAGATTTG |
|  | ZNF558-Rv | GAGTTTCACTCCACGATGACTT |
| *SPATA18* | SPATA18-Fw | GCAGGAAAAGCTAGACTTCTGG |
|  | SPATA18-Rv | TGGCAACTTGCTCAATGAGTTC |
| *MAP2* | MAP2-Fw | CTCAGCACCGCTAACAGAGG |
|  | MAP2-Rv | CATTGGCGCTTCGGACAAG |
| *TUBB3* | TUBB3 -Fw | GCCGAGGAAGAGGGCGAGATG |
|  | TUBB3 -Rv | CAGCTGCGAGCAGCTTCACTTG |
| *PAX6* | PAX6-Fw | TGGGCAGGTATTACGAGACTG |
|  | PAX6-Rv | ACTCCCGCTTATACTGGGCTA |
| *RPS18* | RPS18-Fw | GCGGCGGAAAATAGCCTTTG |
|  | RPS18-Rv | GATCACACGTTCCACCTCATC |
| mtDNA | mtDNA -Fw | CACCCAAGAACAGGGTTTGT |
|  | mtDNA -Rv | TGGCCATGGGTATGTTGTTA |
| nucDNA | nucDNA-Fw | TGCTGTCTCCATGTTTGATGTATCT |
|  | nucDNA-Rv | TCTCTGCTCCCCACCTCTAAGT |
| VNTR | VNTR-Fw | CAGGTGGATGAGGCTCTGAC |
|  | VNTR-Rv | AGGCAGGTTCTGTTGATCGG |
| Albumin | Albumin-Fw | GGAATTCCGCTGTGACCAGA |
|  | Albumin-Rv | ACACCCCTCCATAAGCTCAG |

**Supplementary Table 3. Primer sequence for establishment of CON1_ASTN2^-/-^ cell line**

| Targets | Primers | Sequence (5’→3’) |
| --- | --- | --- |
| Construction of sgRNAs | sgRNA#1-Fw | GAGACCACTTGGATCCGGGCCGGCGGCAAAGGTGACGTTTTAGAGCTAGAAATAGCA |
|  | sgRNA#2-Fw | GAGACCACTTGGATCCGCAGATGACCAGCTCATTTCGTTTTAGAGCTAGAAATAGCA |
|  | sgRNA#3-Fw | GAGACCACTTGGATCCGGGTGTGGAGATTCGCTGTGGTTTTAGAGCTAGAAATAGCA |
|  | sgRNA#4-Fw | GAGACCACTTGGATCCGCTGAGAGGTAGGTGATGAAGTTTTAGAGCTAGAAATAGCA |
|  | sgRNA#5-Fw | GAGACCACTTGGATCCGTTTCCAGCAGCACTGGTGTGTTTTAGAGCTAGAAATAGCA |
|  | sgRNA#universal-Rv | GCCCGGGTTTGAATTCAAAAAAAGCACCGACTCGGTGCCACTTTTTCAAGTTGATAACGGACTAGCCTTATTTTAACTTGCTATTTCTAGCTCTAA |
| *ASTN2* primer for T7E1 assay | ASTN2-Fw | ACTTCCTCAATGGATACCAGCTTC |
|  | ASTN2-Rv | AACAGGCTGAGTCCCTGCACTAC |

**Supplementary Table 4. Predicted off-target sites (Homo sapiens GRCh37/hg19)**

| Coordinates | strand | MM | Target sequence | PAM | position | Gene name |
| --- | --- | --- | --- | --- | --- | --- |
| chr9:116651580-116651602 | + | 0 | ATTTCCAG[CAGCACTGGTGT] | GGG | Exonic | ASTN2 |
| chr2:241204615-241204637 | + | 3 | A**GCC**CCAG[CAGCACTGGTGT] | TGG | Intronic | ANO7 |
| chr5:89488300-89488322 | − | 2 | A**C**TTCCAG[**T**AGCACTGGTGT] | AGG | Intergenic | CTD-2232E5.2 |
| chr4:126974681-126974703 | − | 3 | **C**T**C**TCCAG[**A**AGCACTGGTGT] | GGG | Intergenic | Y_RNA |
| chr14:67483734-67483756 | − | 3 | A**C**T**C**CCAG[**G**AGCACTGGTGT] | GGG | Intronic | TMEM229B |
| chr18:25711715-25711737 | − | 3 | ATTTC**T**A**A**[CAGCA**T**TGGTGT] | AGG | Intergenic | NA |
| chr5:99543668-99543690 | + | 3 | ATTT**AT**AG[CAGCACT**A**GTGT] | TGG | Intergenic | CTD-2151A2.1 |
| chr2:54188811-54188833 | + | 3 | **C**TT**G**CCAG[CAGCACTGG**C**GT] | AGG | Intronic | RNU7-172P |
| chr20:32467889-32467911 | − | 3 | ATTT**A**CAG[CAGCA**G**TG**C**TGT] | TGG | Intronic | NOL4L |
| chrX:138295503-138295525 | + | 3 | ATTT**T**CAG[CA**A**CACTGGT**T**T] | AGG | Intergenic | NA |
| chr12:42075919-42075941 | + | 3 | ATTT**T**CAG[C**T**GCACTGGTG**C**] | AGG | Intergenic | AC023513.1 |
| chrX:135749949-135749971 | + | 3 | ATT**C**CCAG[CAGCA**A**TGGTG**G**] | AGG | Intergenic | MGAT2P2 |
| chrX:135733346-135733368 | − | 3 | ATT**C**CCAG[CAGCA**A**TGGTG**G**] | AGG | Intergenic | MGAT2P1 |
